# Current quality of life questionnaires are not relevant for assessing QOL issues in multiple myeloma patients in the era of modern therapies: results from a survey with myeloma patients and myeloma healthcare professionals

**DOI:** 10.3389/fonc.2025.1656912

**Published:** 2025-10-01

**Authors:** Catherine S. Y. Lecat, Sotirios Bristogiannis, Dipal Mehta, Yadanar Lwin, Joanne Land, Orla McCourt, Emma Dowling, Nuno Correia, Neil K. Rabin, Ke Xu, Jonathan Sive, Xenofon Papanikolaou, Rakesh Popat, Lydia Lee, Annabel McMillan, Eileen M. Boyle, Kwee Yong, Charalampia Kyriakou

**Affiliations:** ^1^ Department of Haematology, University College London (UCL) Cancer Institute, London, United Kingdom; ^2^ Department of Haematology, University College London Hospitals National Health Service (NHS) Foundation Trust, London, United Kingdom

**Keywords:** myeloma, quality of life, PROM (patient reported outcome measurement), healthcare professional (HCP), mutliple myeloma, health related qualitiy of life

## Abstract

As multiple myeloma (MM) patients live longer, maintaining quality of life (QOL) has become an important treatment goal. However, the commonly used general quality of life questionnaires (QOLQs) were developed over 20 years ago. In this survey, 224 MM patients and 48 healthcare professionals (HCPs) were asked to grade the relevance and importance of QOL items from 9 validated, frequently used MM QOLQs. The results from this survey highlighted significant discrepancy between MM patients’ and HCPs’ perception of important and relevant QOL issues. Whilst MM HCPs found all QOL items relevant, the patients reported a proportion of these items being relevant. These were mainly related to physical functioning, social/family wellbeing, pain and fatigue. This real-world survey stressed the need for the development of an updated QOLQ that is relevant to patients and current MM therapies.

## Introduction

Multiple myeloma (MM) is the second most common haematological malignancy characterised by bone marrow proliferation of clonal plasma cells, resulting in anaemia, hypercalcaemia, renal failure and bone destruction. MM patients often experience more symptom burden than patients with some other cancers and have reduced health-related QOL (1). Several validated QOLQs are available to assess QOL in MM patients. Moreover, large clinical trials and real-world studies are increasingly incorporating QOL assessments into their study protocols (2). However, most of these QOLQs such as EORTC-QLQ-C30 and FACT-G are general cancer questionnaires that were designed over 20 years ago, and the MM treatment landscape has since changed dramatically. Aside from newer agents with specific toxicities, the increasing use of consolidation and maintenance combination therapies means that patients often have no treatment-free period. The aim of this survey was to investigate in real-world how relevant and important items from the most commonly used validated QOLQs were for MM patients and MM HCPs.

## Methods

Myeloma patients and HCPs from a single academic MM centre were invited to participate in this specially designed online survey. Demographics, disease characteristics and treatment history were gathered in this anonymised survey. All 172 QOL items from EORTC QLQ-C30, EORTC QLQ-MY20, EORTC QLQ-CIPN20, EQ5D-3L, FACT-G, as well as additional items from the FACT-MM, FACT-N, FACIT-fatigue, FACT-BMT were listed in this survey and participants were asked to grade the relevance of each item in assessing QOL on a five-level Likert scale (not relevant, slightly relevant, relevant, fairly relevant, very relevant). Descriptive statistics (e.g., mean, median, range) were used to summarise the dataset. Weighted mean scores for each item were calculated based on participants’ responses (not relevant scored 1, slight relevant 2, relevant 3, fairly relevant 4, very relevant 5). Depending on where the scores lie in the interval scales, each item was then assigned an overall descriptive equivalent on the Likert scale (3) (See **Supplementary Data**).

## Results

Two hundred- and twenty-four-MM patients (n=224) completed the survey. Their characteristics are shown in **Table 1**. The median age of MM patients was 64 (range 28-82) and the median number of years from diagnosis was 4 (range 1-22). Regarding symptom burden, 38% had bone disease, 29% had spinal disease, 19% had renal impairment and 9% had treatment-related eye problems. A quarter of the patients had newly diagnosed myeloma (25%, n=56). The median number of MM treatment lines was 1.5 (range 1-6). 21% received radiotherapy, 78% received a stem cell transplantation and 27% participated in a MM clinical trial. Patients completed 84% of all survey items (16% were skipped). Forty-eight HCPs completed the survey, including 19 doctors, 5 nurses, 6 pharmacists, 16 MM physiotherapists, 1 occupational therapist, 1 clinical trial practitioner. Nearly 50% (n=23) had over 5 years of experience in the field of MM.

**Table 1:** Patient and healthcare professional characteristics.

Myeloma patient characteristics (n=224)		Frequency (%)
Age group	≤40	7 (3)
	41-50	12 (5)
	51-60	66 (29)
	61-70	72 (32)
	≥71	64 (29)
	Not specified	3 (1)
Gender	Female	89 (40)
	Male	129 (57)
	Not specified	6 (3)
ECOG performance status	0	103 (46)
	1	97 (43)
	2	10 (4)
	3	2 (1)
	Not specified	12 (5)
Disease complications*	Kidney disease	43 (19)
	On haemodialysis	7 (3)
	Bone disease	86 (38)
	Spinal disease	66 (29)
	Treatment-related eye problems	20 (9)
Year(s) of diagnosis	<1	35 (16)
	1-5	88 (39)
	6-10	49 (22)
	>10	19 (8)
	Not specified	33 (15)
ASCT	Yes	169 (75)
	No	47 (21)
	Not specified	8 (4)
Prior CAR-T cell therapy	Yes	2 (1)
	No	213 (95)
	Not specified	9 (4)

Healthcare professional characteristics (n=49)		Frequency (%)
Age group	≤30	4 (8)
	31-40	27 (55)
	41-50	12 (24)
	51-60	3 (6)
	61-70	1 (3)
	Not specified	1 (3)
Gender	Female	40 (82)
	Male	7 (14)
	Not specified	2 (4)
Years of practice in myeloma	<5	23 (48)
	5-10	13 (27)
	>10	10 (21)
	Not specified	2 (4)
Profession	Doctor	19 (39)
	Nurse	5 (10)
	Pharmacist	6 (12)
	Myeloma-specific physiotherapist	16 (33)
	Occupational therapist	1 (3)
	Clinical trial practitioner	1 (3)

ECOG, Eastern Cooperative Oncology Group; ASCT, Autologous Stem Cell Transplant; CAR-T, Chimeric Antigen Receptor T cell therapy. *Multiple options allowed.

Of the 172 HRQOL items, 22 (13%) had an overall description of ‘not relevant’, 105 (61%) were ‘slightly relevant’, 38 (22%) were ‘relevant’ and 7 (4%) were ‘fairly relevant’ in the MM patient group (**Table 2**). None were described as ‘very relevant’. Items that were described as ‘relevant’ or above (relevant, fairly relevant, very relevant) included those related to physical functioning (e.g. able to do strenuous activities and a long walk in EORTC-QLQ-C30), functional well-being (all items in this domain in FACT-G, e.g. able to work and enjoy life) and social/family well-being (6 of the 7 items in this domain in FACT-G, e.g. getting support from family and friends). Symptom items related pain (e.g. bone and back pain in EORTC-QLQ-MY20) and fatigue/energy level (e.g. items in FACIT-fatigue), as well as items regarding infections (e.g. worry about getting infections in FACT-N), memory (e.g. able to remember things in FACT-BMT) and overall health (e.g. rating own health in EQ5D) were described as ‘relevant’ or above. Interestingly, several symptom items such as nausea/vomiting, poor appetite, hair loss, sore eyes/mouth, headaches, and tremors were described as ‘not relevant’ by this patient group.

**Table 2:** Distribution of responses between MM and HCPs cohorts.

Overall description of HRQOL items (n=172)	Frequency of items in MM patient cohort (%)	Frequency of items in HCP cohort (%)
Not relevant	22 (13)	0 (0)
Slightly relevant	105 (61)	0 (0)
Relevant	38 (22)	12 (7)
Fairly relevant	7 (4)	115 (67)
Very relevant	0 (0)	45 (26)

When looking at responses from patient subgroups with renal impairment (n=43), bone disease (n=86) and spinal disease (n=66), the QOL items that these subgroups found relevant largely mirrored the items highlighted in the whole MM patient group. When comparing responses between newly diagnosed and relapsed patients, QOL items related to risk of infections, worries about future health and thoughts regarding their illness had higher weighted mean scores in the newly diagnosed patient group. In the relapsed disease group, items related to the disease’s impact on usual activities (e.g., being able to work), concerns around disease progression, bone pain and tiredness had higher weighted mean scores.

In contrast to the MM patients, MM HCPs graded all 172 items as ‘relevant’ or over. Forty-five items (26%) were ‘very relevant’, 115 (67%) were ‘fairly relevant’, and 12 (7%) items were graded as ‘relevant’. Items considered as ‘very relevant’ included those related to physical and role functioning (e.g. all items in these domains in EORTC-QLQ-C30), all items in the EQ5D, ability to work (e.g. able to work in FACT-G, and concerns about keeping one’s job in FACT-BMT), as well as symptom items on pain and fatigue.

## Discussion

As myeloma patients survive longer with improved therapies, maintaining QOL is a significant treatment endpoint. Increasingly, researchers are incorporating appropriate QOL measures as primary endpoints now that we have a plethora of therapeutics options. This real-world survey was one of few studies that interrogated which items MM patients found relevant from commonly used QOLQs. With its simple design, uptake of this study was good and it included patients with a wide age range, time from diagnosis, different stages of disease, different symptom burden at presentation or relapse and variable therapy lines exposure. Despite it being a long survey of over 172 questions, it was feasible to conduct, with only 16% of items in the survey skipped by patients.

Apart from doctors, various HCPs within the multidisciplinary care team also participated in the survey, allowing us to understand how their views differ from those of the patients. It was surprising to find out that MM patients found the majority (74%) of QOL items as ‘irrelevant’ or ‘slightly relevant’. Only 26% of items were identified and reported as ‘relevant’ or above. Most of these questionnaires were designed and validated when traditional chemotherapeutic regimens were main treatment options for MM. For example, EORTC QLQ-C30 and -MY20 were developed in 1993 and 1996 respectively, long before proteasome inhibitors, anti-CD38, IMiDs, bispecific T cell engagers and CAR T Cell therapies became new standard of care for treating MM. Patients are also now more likely to be on continuous treatment until disease progression without any treatment breaks, leading to cumulative toxicities. Multiple newer targeted agents are being increasingly prescribed in combination. These have unique side effect profiles, which may not be accurately assessed by current QOLQs. Despite this, researchers continue to draw conclusions from these existing QOLQs in clinical trials that test newer agents (4, 5). There have been attempts to develop new PRO tools such as MyPOS (6) and HM-PRO (7), which have included important additional items related to sex life, information needs and confidence in the clinical team. However, despite the rigorous process involved in developing these newer tools, they remain underused compared to older ones. This survey highlighted the need to update and validate new representative modules for more reliable, harmonised QOL reporting.

In contrast to MM patients, MM HCPs considered all QOL items relevant for assessing patients’ QOL. The discrepancy reflects the difference in the perceptions of QOL priorities between MM patients and HCPs. The results from this survey could help and alert HCPs to focus their attention, resources and treatment decision plans on relevant items highlighted by patients. For example, MM patients graded fatigue, infection and pain as relevant QOL issues. Pain from bone disease is a very common feature in myeloma. Optimal pain control is challenging and likely involves multiple strategies combining targeted chemotherapy, radiotherapy, and pain medications in a personalised and multidisciplinary approach (8). Spinal bracing, kyphoplasty and vertebroplasty can be considered for those with painful vertebral fractures but more studies are needed as data regarding clinical outcomes and QOL on patients with spinal interventions is scarce. Fatigue is another common, distressing symptom graded as very relevant by MM patients. One way to tackle this problem would be to promote physical activity, which has been shown to improve not only fatigue but also physical functioning and HRQOL in cancer patients (9). Tailored exercise programmes have been shown to be safe and effective in MM and could be incorporated into the pre- and rehabilitation pathways (10). Infections remain a major concern for both patients and HCPs, particularly for those receiving bispecific T cell engagers and CAR T cell therapy. Clinicians should follow up-to-date guidance on antimicrobial prophylaxis and infection management in these patients (11). They should also explore ways to improve treatment tolerability, such as better infection risk assessment and using frailty-adjusted dose modification (12). Overall, having harmonised criteria for QOL will support MM patients and empower their HCPs in treatment management targeting superior QOL outcomes.

The limitations of this study include its single-centred design and the fact that it was not powered for subgroup analysis. Although it included a broad MM population, a small percentage of patients received new therapies such as CAR-T therapy and bispecific T cell engagers. Nonetheless, the feedback from this real-world survey highlighted the need for more relevant QOLQs to access QOL in modern-day MM patients. The lack of relevant questionnaires may explain their limited use in routine clinical environments. Further qualitative studies are needed to ensure new concepts and treatment side effects are included in future QOL tools.

## Data Availability

The original contributions presented in the study are included in the article/**Supplementary Material**. Further inquiries can be directed to the corresponding author.
