# Supplementary material for: Current quality of life questionnaires are not relevant for assessing QOL issues in multiple myeloma patients in the era of modern therapies: results from a survey with myeloma patients and myeloma healthcare professionals
Source: Front Oncol. 2025 Oct 1;15:1656912. doi: 10.3389/fonc.2025.1656912 (PMC12520875; doi:10.3389/fonc.2025.1656912)
Supplement: Supplementary file 1 [file Table1.docx]

**Interpreting Likert scale results using descriptive equivalent**

1. **Assign a score to each Likert scale response**

| *Likert scale response* | *Score* |
| --- | --- |
| Not relevant | 1 |
| Slightly relevant | 2 |
| Relevant | 3 |
| Fairly relevant | 4 |
| Very relevant | 5 |

1. **Determine the range and the interval**

Range = highest score – lower score = 5 – 1 = 4

Interval = highest score / range = 5 / 4 = 0.80

1. **Work out the interval scales for each Likert scale response**

E.g. For ‘not relevant’ which has a score of 1, the interval scale would be 1 to (1 + interval = 1 + 0.80 = 1.80). For ‘slightly relevant’ which has a score of 2, the interval scale would be 1.81 to (1.80 + interval = 1.80 + 0.80 = 2.60) and so on:

| *Likert scale response* | *Interval scales* |
| --- | --- |
| Not relevant | 1.00 – 1.80 |
| Slightly relevant | 1.81 – 2.60 |
| Relevant | 2.61 – 3.40 |
| Fairly relevant | 3.41 – 4.20 |
| Very relevant | 4.12 – 5.00 |

1. **Calculate the weighted mean score**

For each QoL item, work out the weighted mean score by adding up all the scores, then decided by the number of responses for that item.

Weighted mean score = Total score of the QoL item / no. of responses

1. **Assign a descriptive equivalent to each QoL**

Based on where the weighted mean score lies in the interval scales, assign the descriptive equivalent to the QoL item.

E.g. Item X has a weighted mean score of 3.29. This value lies within the interval scale for ‘relevant’ which is 2.61 – 3.40. Thus, item X has a descriptive equivalent of ‘relevant’.
